# Comparison of mechanical and manual bone marrow puncture needle for intraosseous access; a randomized simulation trial

**DOI:** 10.1186/s40064-015-0982-y

**Published:** 2015-05-02

**Authors:** Fumihiro Ohchi, Nobuyasu Komasawa, Ryosuke Mihara, Toshiaki Minami

**Affiliations:** Department of Anesthesiology, Osaka Medical College, Daigaku-machi 2-7, Takatsuki, Osaka 569-8686 Japan

**Keywords:** Intraosseous access, Manual bone marrow puncture needle, Mechanical bone marrow puncture needle, Simulation

## Abstract

**Background:**

During resuscitation, when it is difficult or impossible to establish peripheral venous access, intraosseous route (IO) is considered as an alternative to a central venous line. However, it is sometimes difficult for obtain IO access with conventional manual bone puncture needle. Recently, powered mechanical bone marrow needle was developed. We compared the performance of the manual and mechanical bone marrow puncture needle for adult, child and infant simulation.

**Methods:**

22 anesthesiologists, who has never used bone marrow puncture needle, performed manual (Dickman™, Cook Medical) or mechanical (EZ-IO™, Teleflex) bone marrow puncture to simulated adult, child and infant tibia. Puncture success rate, insertion time, and subjective difficulty utilizing visual analogue scale was assessed.

**Results:**

In adult settings, with the manual bone marrow needle, only 3 of 22 participants could succeed in the IO route keep, while all participants did in the mechanical bone marrow puncture needle (P < 0.001). In child and infant settings, all trials were successful in both manual and mechanical bone marrow puncture needles (P = 1.00). In adult simulations, IO insertion took significantly longer with manual bone marrow puncture (54.8 ± 15.8 s) than without compressions (3.7 ± 2.1 s; P < 0.001). In child and infant simulations, the IO insertion time was significantly smaller in mechanical trials than in manual ones (child simulation; manual 9.3 ± 4.6 s, mechanical 2.2 ± 0.8 s, P < 0.001, infant simulation; manual 2.0 ± 1.1 s, mechanical 1.5 ± 0.8 s, P = 0.003).

Although the VAS score was not significantly higher with manual trials than in mechanical trials among the three simulations (adult simulation, P < 0.001, child simulation, P < 0.001, infant simulation P = 0.006).

**Conclusions:**

We conclude that in simulations managed by anesthesiologists who had no clinical experiences with bone marrow puncture, the mechanical bone puncture needle performed better than the manual one for emergency IO route access.

## Introduction

The European Resuscitation Council (ERC) cardiopulmonary resuscitation (CPR) guidelines emphasize the importance of minimizing chest compression interruptions to maximize coronary and cerebral perfusion pressure (Nolan et al. 2010). The guidelines also suggest that skilled rescuers should be able obtain rapid and reliable airway or vascular access without interrupting chest compressions (Neumar et al. 2010; Deakin et al. 2010). However, keeping definite vascular access is often difficult for cardiopulmonary collapsed patients. When it is difficult or impossible to establish peripheral venous access, intraosseous route (IO) is considered as an alternative to a central venous line (Blumberg et al. 2008).

Bone marrow puncture needle insertion for IO vascular access is a standard procedure used, especially in paediatric resuscitation (Fiorito et al. 2005), and IO devices has expanded its role to include resuscitation in patients of all ages (Glaeser 1993). However, it is sometimes difficult to perform IO rapidly and precisely with conventional manual bone marrow puncture needle. Furthermore, managing resuscitation can be challenging if the essential drugs or infusion cannot be administered (Vidal et al. 1993).

Recently, the mechanical bone marrow needle EZ-IO™ (EZ-IO, Teleflex, Pennsylvania, U.S.A.) has been developed and its feasibility has been reported (Gillum & Kovar 2005; Cooper et al. 2007). However, the utility for IO access with manual or mechanical bone marrow puncture needle has not been compared yet. As it is unethical to perform the validation of these needles in clinical settings first, we decided to perform the comparison between manual and mechanical bone marrow puncture needle utilizing simulation.

The present study aimed to determine which of the two devices would improve IO access faster and definitely. To this end, we compared the performance of the manual and mechanical bone marrow puncture needle for adult, child and infant simulation.

## Materials and methods

This study was judged as no need for registration by the institutional review board of Osaka Medical College because this study does not include any patient or volunteer intervention. On 31th, January 2015, 22 anesthesiologists, who has never used bone marrow puncture needle, were recruited during peripheral nerve block training simulation course at Osaka Medical College. Selected participants had 9.9 ± 5.9 years of clinical experience in anesthesia.

**Figure 1:**
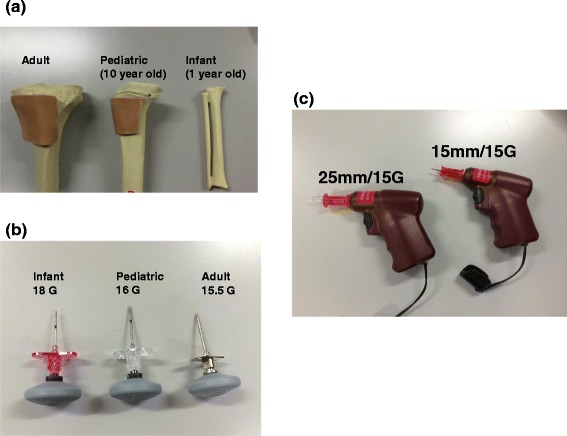
Simulated adult, child, and infant tibias **(a)**, and manual **(b)** and mechanical **(c)** bone marrow puncture needle.

**Figure 2:**
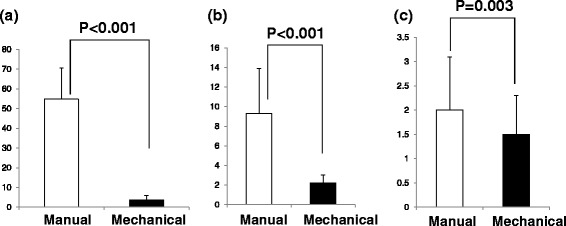
Intraosseous (IO) insertion time with manual and mechanical bone marrow puncture needle. **(a)** adult simulation, **(b)** child simulation, and **(c)** infant simulation. Manual: IO insertion with manual bone marrow puncture needle; Mechanical: IO insertion with mechanical bone marrow puncture needle. *P < 0.05 compared to without chest compression.

The Proximal Tibia with skin patch® for adult simulation, Pediatric Tibia with skin patch® for 10-year-old child, and Tibia Fibula combination® simulated for 1 year infant (Vidacare, San Antonio, U.S.A.) was used to perform manual or mechanical bone marrow needle puncture (Figure 1). These simulated tibia were made of polyurethane resin. Dickman bone marrow infusion needle® (Cook Medical, Indiana, U.S.A) were used for Manual bone marrow puncture needle, which is shown in Figure 2 (adult 15.5G 3 cm, child 16G 3cm, infant 15.5G 2.5 cm). Mechanical bone marrow needle (Vidacare, San Antonio, U.S.A.) was used 25 mm/15G for adult and 15 mm/15G for child and infant simulation (Figure 1c).

The simulated bone was placed on a hard, flat table. Each participant was given 5 minutes for manual and mechanical bone marrow needle puncture. Each participant was instructed to hold the manual or mechanical bone marrow needle and penetrate the bone safely and rapidly as possible, but not to penetrate to the other side of the bone. The instructor did not give any advice during the trial. Insertion started when the participant picked up the manual or mechanical bone marrow puncture needle and ended at the point of bone penetration. The puncture success or failure (could not penetrate the outer surface of the bone to inner lumen) was judged by the same person. Trial which was penetrated to the counter side of the bone was considered as failure. The limitation is 60 seconds and if the participants could not keep IO within 60 seconds it is considered for failure. Insertion times were recorded for 60 second if they could not keep IO within this time period. At the end of the study, participants rated the difficulty of using both bone marrow needles for IO route keeping on a visual analog scale (VAS) from 0 mm (extremely easy) to 100 mm (extremely difficult) (Komasawa et al. 2011).

Results obtained from each trial were compared paired Students t test for insertion times and VAS, and Fisher’s exact test for the success rate. Data are presented as mean ± SD. P < 0.05 was considered statistically significant.

In this study, each participant perform manual and mechanical IO on three simulated bones (infant, child, adult). This increase the risk of learning curve effect. Thus, we performed this trial as a randomized cross-over design to minimize the learning curve effect. The order of six intervention was determined for each participant by random table number by computer (720 patterns). Each participant performed six interventions in a different sequence (Komasawa et al. 2013).

Results of a ten-doctor preliminary study showed that the time required for successful insertion of the mechanical bone marrow puncture was approximately 5.2 ± 1.3 s. To detect a 33% difference, we estimated that 18 participants would be adequate for two independent groups, whereby α = 0.05 and β = 0.2.

## Results

### IO insertion success with manual and mechanical bone marrow puncture needle

Numbers of successful IO for both needles are displayed in Table 1. In adult settings, with the manual bone marrow needle, only 3 of 22 participants could succeed in the IO access keep, while all participants did in the mechanical bone marrow puncture needle. The success rate was significantly higher in mechanical bone marrow puncture needle trial than in manual one (P < 0.001).

**Table 1:** **Bone marrow puncture success rates within 1 minute for manual and mechanical bone marrow puncture needle**

	**Manual BMPN**	**Mechanical BMPN**	***P*** **-value (Fisher’s exact test)**
Adult	3/22	22/22	<0.001
Child	22/22	22/22	1.00
Infant	22/22	22/22	1.00

BMPN: Bone marrow puncture needle.

Values are presented as number of participants who achieved successful intubation/number of participants who attempted bone marrow puncture.

In child and infant settings, all trials were successful in both manual and mechanical bone marrow puncture needles, which did not show significant difference (P = 1.00).

### IO Insertion time with the manual and mechanical bone marrow puncture needle

IO insertion times are shown in Figure 2. In adult simulations, IO insertion took significantly longer with manual bone marrow puncture (54.8 ± 15.8 s) than without compressions (3.7 ± 2.1 s; *P* < 0.001) (Figure 2a). In child and infant simulations, the IO insertion time was significantly smaller in mechanical trials than in manual ones (child simulation; manual 9.3 ± 4.6 s, mechanical 2.2 ± 0.8 s, P < 0.001, infant simulation; manual 2.0 ± 1.1 s, mechanical 1.5 ± 0.8 s, P = 0.003).

### VAS scores for IO insertion with manual and mechanical bone marrow puncture needle

**Figure 3:**
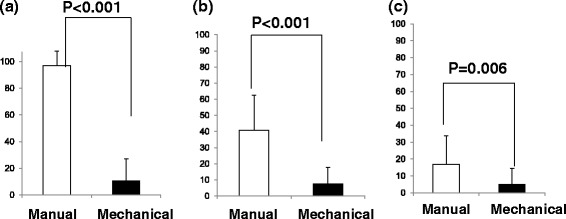
Visual analog scale for Intraosseous (IO) insertion time with manual and mechanical bone marrow puncture needle. **(a)** adult simulation, **(b)** child simulation, and **(c)** infant simulation. Manual: IO insertion with manual bone marrow puncture needle; Mechanical: IO insertion with mechanical bone marrow puncture needle. *P < 0.05 compared to without chest compression.

As shown in Figure 3, although the VAS score was not significantly higher with manual trials than in mechanical trials among the three simulations (adult simulation, P < 0.001, child simulation, P < 0.001, infant simulation P = 0.006).

## Discussion

Current ERC-CPR guidelines emphasize the delivery of continuous chest compression with as few interruptions as possible, including pauses for vascular access, defibrillation, or management efforts (Nolan et al. 2010; Okada et al. in press). Several studies have shown that prolonged interruption of chest compressions is associated with both decreased coronary and cerebral perfusion and reduced venous return to the heart, resulting in low survival rates and impaired post-resuscitation myocardial function (Deakin et al. 2010). During cardiac arrest, after beginning chest compression and attempting defibrillation for identified VF or pulseless VT, providers can establish intravenous (IV) or IO access. The primary purpose of IV/IO access during cardiac arrest is to provide drug therapy. Several clinical studies reported data suggesting worsened survival for every minute that antiarrhythmic drug delivery was delayed (Dorian et al. 2002; Kudenchuk et al. 1999).

IO cannulation provides access to a noncollapsible venous plexus, enabling drug delivery similar to that achieved by peripheral venous access at comparable doses. Various clinical trials suggest that IO access can be established efficiently; is safe and effective for fluid resuscitation, drug delivery, and blood sampling for laboratory evaluation; and is attainable in all age groups (Guy et al. 1993; Banerjee et al. 1994; Stone et al. 2007). It is reasonable for providers to establish IO access if IV access is not readily available in the ERC guidelines (Deakin et al. 2010).

The IO approach is a very fast, simple infusion technique but non-negligible number of IO insertion failure even by trained emergency physicians remain (Simmons et al. 1994; Moscati & Moore 1990). One possible reason of high failure rate was caused mainly by the inability to control the path of the catheter. With manual bone marrow puncture needle, penetration of the bone needs relatively massive force especially in adult bones and it is also difficult to confirm the correct placement of the needle (Horton & Beamer 2008). Compared to manual bone puncture needle, the mechanical bone marrow puncture needle not only provide rapid puncture of the bone with powered drill but also prevent the excessive penetration of the bone with relatively short needles. Though there have been several reports about the utility of mechanical bone marrow puncture needle (Gillum & Kovar 2005; Cooper et al. 2007; Horton & Beamer 2008), our simulation study is the first direct comparison between manual and mechanical bone marrow puncture needles.

In our study, only three doctors could puncture adult bone within 60 seconds, while all participants could with mechanical one rapidly. It is often difficult to keep definite IO access with mechanical bone marrow needle in adult patients during emergency. Our result suggests the utility of powered mechanical needle for IO access to puncture the hard bone rapidly and effectively, especially for adults. For child and infant simulations, although all participants could puncture the bone regardless of the needle type, the insertion time was significantly shorter in mechanical trial than in manual ones. As IO access is usually considered in emergency situations, it is important to secure rapidly as possible. From the viewpoint of IO access puncture time, mechanical bone marrow needle may be useful in various emergency situations of all ages. Furthermore, application of mechanical bone marrow needle is useful in difficult intravenous access in critical care or perioperative management.

This study has several limitations worth noting. First, the simulated tibia is different from that of real patients in some points. The simulated tibia do not contain other part of the human anatomy except tibia and surrounding structures. In real patients, we can confirm the back-flow of bone marrow blood. Utilizing a simulator with surrounding anatomy and blood, we may evaluate the miss rates more clearly. Furthermore, we could not get simulated infant tibia with skin, which made the comparison to child or adult difficult. Second, we could not simulate the efficacy of IO access evaluation during chest compression. Third, bone marrow puncture were performed on manikin, which leads to shorter airway intervention times than that required for actual patients (Komasawa et al. 2014). Based on our simulation studies, clinical comparison of manual and mechanical bone marrow puncture needle is needed in the future study.

We conclude that in simulations managed by anaesthesiologists who had no clinical experiences with bone marrow puncture, the mechanical bone puncture needle performed better than the manual one for emergency IO route access.

## References

[CR1] Banerjee S, Singhi SC, Singh S, Singh M (1994). The intraosseous route is a suitable alternative to intravenous route for fluid resuscitation in severely dehydrated children. Indian Pediatr.

[CR2] Blumberg SM, Gorn M, Crain EF (2008). Intraosseous infusion: a review of methods and novel devices. Pediatr Emerg Care.

[CR3] Cooper BR, Mahoney PF, Hodgetts TJ, Mellor A (2007). Intra-osseous access (EZ-IO) for resuscitation:UK military combat experience. J R Army Med Corps.

[CR4] Deakin CD, Nolan JP, Soar J, Sunde K, Koster RW, Smith GB, Perkins GD (2010). European Resuscitation Council Guidelines for Resuscitation 2010 Section 4. Adult advanced life support. Resuscitation.

[CR5] Dorian P, Cass D, Schwartz B, Cooper R, Gelaznikas R, Barr A (2002). Amiodarone as compared with lidocaine for shock-resistant ventricular fibrillation. N Engl J Med.

[CR6] Fiorito BA, Mirza F, Doran TM, Oberle AN, Cruz EC, Wendtland CL, Abd-Allah SA (2005). Intraosseous access in the setting of paediatric critical care. Pediatr Crit Care Med.

[CR7] Gillum L, Kovar J (2005). Powered intraosseous access in the prehospital setting: MCHD EMS puts the EZ-IO to the test. J Emerg Med Ser.

[CR8] Glaeser PW (1993). Hellmich, Szewczuga D, Losek JD, Smith DS. Five-year experience in prehospital intraosseous infusions in children and adults. Ann Emerg Med.

[CR9] Guy J, Haley K, Zuspan SJ (1993). Use of intraosseous infusion in the pediatric trauma patient. J Pediatr Surg.

[CR10] Horton MA, Beamer C (2008). Powered intraosseous insertion provides safe and effective vascular access for paediatric emergency patients. Pediatr Emerg Care.

[CR11] Komasawa N, Ueki R, Fujii A, Samma A, Nakagawa M, Nishi S, Kaminoh Y (2011). Comparison of Laryngeal Mask Supreme® and Softseal® for airway management in Several Positions. J Anesth.

[CR12] Komasawa N, Ueki R, Yamamoto N, Nishi S, Kaminoh Y, Tashiro C (2013). Comparison of Pentax-AWS Airwayscope, Airtraq and Miller laryngoscope for tracheal intubation by novice doctors during infant cardiopulmonary resuscitation simulation: a randomized crossover trial. J Anesth.

[CR13] Komasawa N, Fujiwara S, Haba M, Mihara R, Minami T. Comparison of Quick Track and Melker for emergent invasive airway management during chest compression: A crossover simulation trial. Eur J Anaesthesiol. 2014 Aug 8. [Epub ahead of print]. 10.1097/EJA.0000000000000144 25111538

[CR14] Kudenchuk PJ, Cobb LA, Copass MK, Cummins RO, Doherty AM, Fahrenbruch CE, Hallstrom AP, Murray WA, Olsufka M, Walsh T (1999). Amiodarone for resuscitation after out-of-hospital cardiac arrest due to ventricular fibrillation. N Engl J Med.

[CR15] Moscati R, Moore GP (1990). Compartment syndrome with resultant amputation following intraosseous infusion. Am J Emerg Med.

[CR16] Neumar RW, Otto CW, Link MS, Kronick SL, Shuster M, Callaway CW, Kudenchuk PJ, Ornato JP, McNally B, Silvers SM, Passman RS, White RD, Hess EP, Tang W, Davis D, Sinz E, Morrison LJ (2010). Part 8: adult advanced cardiovascular life support: 2010 American Heart Association Guidelines for Cardiopulmonary Resuscitation and Emergency Cardiovascular Care. Circulation.

[CR17] Nolan JP, Soar J, Zideman DA, Biarent D, Bossaert LL, Deakin C, Koster RW, Wyllie J, Böttiger B (2010). European Resuscitation Council Guidelines for Resuscitation 2010: Section 1. Executive summary. Resuscitation.

[CR18] Okada D, Komasawa N, Fujiwara S, Minami T. Comparison of tube-guided and guideless videolaryngoscope for tracheal intubation during chest compression in a manikin: a randomized crossover trial. J Anesth in press 10.1007/s00540-014-1936-1 25348686

[CR19] Simmons CM, Johnson NE, Perkins RM, van Stralen D (1994). Intraosseous extravasation complication reports. Ann Emerg Med.

[CR20] Stone MB, Teismann NA, Wang R (2007). Ultrasonographic confirmation of intraosseous needle placement in an adult unembalmed cadaver model. Ann Emerg Med.

[CR21] Vidal R, Kissoon N, Gayle M (1993). Compartment syndrome following intraosseous infusion. Paediatrics.

